# Graphical and interactive spatial proteomics image analysis workflow

**DOI:** 10.46471/gigabyte.186

**Published:** 2026-06-22

**Authors:** Pritpal Singh, Jocelyn H. Wright, Kimberly S. Smythe, Bryce Fukuda, Ling-Hong Hung, Cecilia C. S. Yeung, Ka Yee Yeung

**Affiliations:** ^1^ School of Engineering and Technology, University of Washington Tacoma, WA, USA; ^2^ Translational Science and Therapeutics Division, Fred Hutchinson Cancer Center, Seattle, WA, USA

## Abstract

Spatial proteomics provides a spatially resolved view of protein expression and localization within cells and tissues by mapping the location and abundance of proteins. There is a need for fully-integrated end-to-end imaging workflows for spatial proteomic analysis that are flexible, reproducible, and support graphical and interactive visualizations. We present a modular and interactive spatial proteomic image analysis workflow with individual containerized steps that empowers biomedical researchers to reproducibly execute and customize complex analyses. Our workflow consists of cell segmentation, unsupervised clustering with optional batch correction, validation of clusters on the image, and cell type clustering results visualization. A form-based graphical interface can be utilized to execute and customize multi-step workflows with a single click or interactively adjust image processing steps within the workflow, apply workflows to various datasets, and modify input parameters as needed. We illustrated the functionality of our workflow using human normal tonsil and colorectal cancer tissues stained by high-plex immunohistochemistry.

## Introduction

The advent of high-plex spatial proteomics allows interrogation of patient tissue samples with over fifty-five antibody protein markers simultaneously, allowing for novel spatial biomarkers (e.g., specific cell type interactions, protein marker co-expression, or specific morphologies) to be identified that can predict disease. Techniques such as co-detection by indexing (CODEX), run on the PhenoCycler-Fusion (PCF) platform, are currently among the leading spatial proteomics imaging approaches. Analysis of protein staining must consider not only expression level of a biomarker as is the case with spatial transcriptomics, but also the location of the staining (nuclear, cytoplasmic, or membranous), the staining pattern (dot like, linear, contiguous, diffuse and homogenous), and integration of cell morphology to allow for correct interpretation. These multi-modal analytical workflows often consist of analysis steps performed using different analysis tools or components that are not interoperable.

Numerous software platforms can be used to analyze spatial proteomics imaging data, but they often come with limitations that pose challenges for users. A key limitation of existing approaches is the lack of an end-to-end software solution that enables users to interact with graphical visualizations and provide domain knowledge to guide the analyses. Most workflow execution engines focus on automating, orchestrating, and optimizing the execution of complex tasks but often lack native support for graphical outputs. However, support for interactive graphical analysis is essential to facilitate interpretation of imaging data. Another desirable feature is the flexibility to integrate open-source tools that are widely adopted by the bioimage community.

Our work is in contrast to Enable Medicine [1], a commercial platform that offers graphical output, consistency, and reproducibility of analysis when images are uploaded to their proprietary server. In addition to the lack of support for on-prem analysis, its closed-source platform does not allow customization without a premium license, so it is not possible to integrate widely used bioimage tools like QuPath [2] or customized segmentation methods. Data output is also not flexible. Another example, SPEX (Spatial Expression Explorer) is an open-source modular analysis platform with a graphical user interface that supports spatial proteomics and spatial transcriptomics workflows [3]. However, the support for interactive analysis of graphical output and flexibility of integrating commonly used graphical tools are limited. Software solutions in the form of data science notebooks, such as SPACEc [4] have also been proposed. However, it is not designed as a plug-and-play workflow for biomedical users, and it is non-trivial for biomedical users to create and customize data science notebooks. It is unclear how interactive third party tools, such as QuPath which is a cross-platform Java application, can be supported within their provided Python notebooks. In contrast, containerized modules in our end-to-end workflow can span across different computing environments, support graphical user input and output.

Here, we present a modular and interactive graphical platform that empowers biomedical researchers to reproducibly execute complex spatial proteomic analysis in an automated end-to-end workflow that easily integrates multiple open-source software tools and custom scripts through containerization. Containerization enables reproducibility and portability by bundling a software application, along with all its dependencies and specific configurations, into a single file known as a container image. Our version-controlled software containers allow our pipeline to run consistently across various computing environments. The end-to-end workflow consists of cell segmentation, cell-level normalization across biomarkers, unsupervised clustering, validation, and interactive graphical visualization of results. The workflow is customizable to allow changes in user defined analysis parameters or inclusion of additional analysis modalities for spatial analysis of cell types.

## Analytical workflow and case study

We developed a spatial proteomics imaging workflow and applied it to two tissues with dissimilar architecture and cell types, namely human normal tonsil and colorectal cancer. The images were stained by high-plex immunohistochemistry using PCF (PhenoCycler-Fusion). PCF offers deep insights into tissue architecture, cellular organization, and the role of the microenvironment in diseases like cancer. Our previous analysis of PCF data using the open-source programs, QuPath [2] to visualize and segment the data and CytoMAP [5] for clustering analysis, were successful but cumbersome to move the data back and forth between the two programs and not always reproducible. Analysis on the Enable Medicine platform was faster and more consistent, but data output was not flexible. In addition, analysis on Enable Medicine required images to be uploaded to their proprietary server which was time consuming and expensive to store image data and perform analyses. Most importantly, sharing data with third party servers complicates data management, compliance, privacy, and security issues.

**Figure 1.**
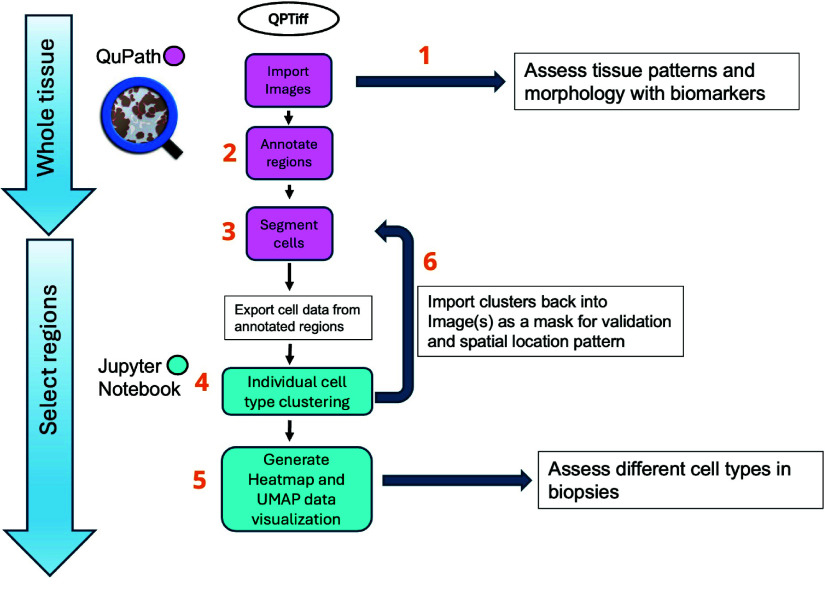
A schematic diagram of an analytical workflow using QuPath for image quality review, region annotation and cell segmentation and a Python Jupyter Notebook to perform unsupervised cluster analysis of cell data for export and visualization as image overlays, Heatmap and UMAP displays.

Building on our previous experience working with PCF data, we designed and developed a multi-step analytical workflow leveraging open-source programs and protocols (Figure 1). This workflow is designed to allow customization by addition/modification of analysis steps, parameters, and output via modular containers. As outlined in Figure 1, steps of the workflow include (1) visualization of the stained image for assessing quality control of individual stains, (2) annotating regions of interest, (3) segmentation of cells into cell data units, (4) exporting these data and performing unsupervised clustering of cell types, (5) generation of data summaries such as heatmaps and dimension reduction techniques (e.g., UMAP) to facilitate cell-type annotation and distribution between samples, (6) creation of color mappings to be superimposed onto the image for visual validation and viewing of spatial patterns in biopsies. Our goal was to make this validated yet cumbersome workflow to function in an end-to-end platform without piecewise data transfers between different software tools.

## Implementation

### Overview

**Figure 2.**
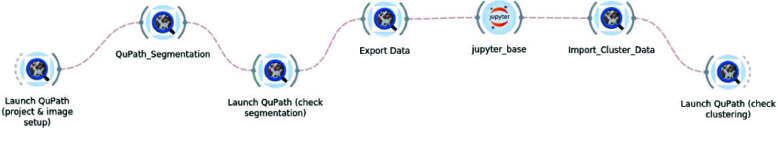
A screenshot showing the graphical containerized workflow consisting of modular widgets.

We implemented an automated workflow of analytical protocols adopted in our CLIA accredited laboratory at the Fred Hutchinson Cancer Center. Our spatial proteomics analysis workflow, as illustrated in Figure 2, supports the execution of graphical applications (QuPath) within a containerized environment and allows users to view and interact with image(s) and QC markers as if these are standalone desktop applications. QuPath is an open source platform that is widely used by the bioimage community. Using this workflow, users can select regions on the image(s) and perform cell segmentation within a QuPath project using an optimized version of the StarDist algorithm [6], then export the cell object data containing *x*, *y* coordinates and mean cell fluorescence pixel intensity (MFI) values for each biomarker into a csv file. The exported cell data can then be used to perform unsupervised clustering of cell types using the Leiden algorithm and visualizing the clustering results as Uniform Manifold Approximation and Projections (UMAP) and heatmaps using Jupyter Notebooks and python libraries. Lastly, the workflow allows importing clustering data back into QuPath to make colormaps to review with the original image as an overlay to validate the results. All analysis steps in the workflow can be executed either end-to-end with a single click or run individually in an interactive manner.

### Graphical widgets

Our spatial proteomics analysis workflow leverages the Biodepot-workflow-builder (Bwb) platform [7, 8], a desktop application that supports interactive graphical output where each task (or module) in the workflow is represented by a graphical widget. These widgets are containerized using Docker containers and linked to other widgets to produce a full workflow. Users can interact with each widget and adjust the parameters using a form-based user interface. Users can also incorporate new tools and/or scripts into their workflow.

To perform the analysis, users can interact with each widget individually or run them as part of the end-to-end workflow. Each widget is associated with a Dockerfile that is used for containerization. In particular, the **Launch_QuPath** widget is used to launch a standalone instance of QuPath within Bwb for managing the QuPath project, performing the analysis, and visualizing the results. This widget has two optional parameters to specify: the QuPath project file and an image to open when QuPath is launched. If these parameters are not specified, QuPath will launch without a project open by default.

### Cell segmentation

The **segmentation** widget allows users to run the StarDist segmentation algorithm within QuPath on the entire image or an individual tissue core in a tissue microarray (TMA) by specifying a parameter of the widget. StarDist is a deep learning-based image segmentation method specifically designed for biological images, such as those obtained from microscopy. This widget depends on my_stardist.groovy script and stardist_cell_seg_model.pb StarDist model file. The two required fields of this widget include qpProjFile and image_to_segment. Users can optionally specify image resolution and region of interest (ROI) to segment via the image_resolution and core_to_segment parameters. By default, the widget will segment all the tissues in the specified image with image resolution set to 0.5. Default settings are tailored for PCF QPTIFF images, however parameters can be adjusted for analysis of Keyence QPTIFFS, as well as images with OME-TIFF format from Lunaphore COMET platforms.

### Clustering

The **jupyter_base** widget launches the BWBQuPathClustering.ipynb Jupyter Notebook that runs the Leiden unsupervised clustering algorithm on the QuPath cell data in the all-cell-measurements.csv file, exported via the **Export_Image_Data** widget. The cell-level biomarker data is normalized prior to clustering using a three-step procedure. Each biomarker expression first undergoes an arcsinh (inverse hyperbolic sine) transformation with a cofactor equal to the 20th percentile of its expression distribution. The transformed data then gets *z*-standard normalization across all cells for each biomarker, followed by *z*-standard normalization across all biomarkers within each cell. This sequential normalization process reduces the influence of extreme values, harmonizes biomarker scales, and ensures that each cell contributes comparably to downstream Leiden clustering.

When clustering cells drawn from multiple tissue regions or multiple images, we observed batch-associated structures in the biomarker profiles, leading the Leiden algorithm to form clusters that were largely segregated by region rather than by underlying biological similarity. To correct for these region-specific batch effects, our pipeline allows users to apply ComBat [9], an empirical Bayes method that adjusts for systematic shifts in feature means and variances across predefined batches. We adopted the Scanpy implementation of ComBat [10] (scanpy.pp.combat), with the region annotation provided as the batch key. This procedure harmonizes biomarker distributions across regions while preserving biological variability, enabling cells from different regions and images to co-cluster when they exhibit similar expression patterns. The cells belonging to different regions and images would then eliminate technical differences between regions to produce more coherent and interpretable cell groups generated from Leiden clustering. This batch correction step using ComBat is optional and can be disabled in the Jupyter notebook widget.

Our Python notebook then depicts the clustered data using UMAPs (Uniform Manifold Approximation and Projection) and heatmaps. Clusters in the UMAP plot can be used to identify groups of cells that belong to the same cell type based on their patterns of biomarker expression. The UMAP is generated using the Python Scanpy package, utilizing the scanpy.pp.neighbors function to generate neighbors, with n_neighbors. The scanpy.tl.umap function is used to generate the UMAP plot with min_dist parameter. The default values for n_neighbors and min_dist are 30 and 0.0001 respectively but can be configured in the notebook. When multiple regions or images are selected, an additional UMAP colored by their image sources or regions is generated. The interactive unsupervised clustering heatmap is plotted using the Python graphing library Plotly and it uses rows to represent biomarkers and columns to represent clusters. The final output of the notebook is the clustering data in comma-separated values (CSV) format, written to leiden_clustering_export.csv in the clustering_data_export directory. This CSV file can be exported and sorted using standard spreadsheet programs as well.

### Overlay of clustering results with images

The **Import_Cluster_Data** widget imports the clustering data from the csv files in the clustering_data_export directory into QuPath to generate colormap overlays onto the original image using the import_clusters.groovy script. Each cluster is assigned a unique color. The spatial arrangement of these clusters is displayed directly on the tissue image which allows to see whether certain clusters localize to specific regions, providing insights into tissue architecture or pathology. The color mappings are designed to have the same color as in corresponding UMAPs to allow easy comparison of cell type cluster within UMAP and spatial location of associated cells within the image. The one and only required parameter for this widget is the qpProjFile to specify the QuPath project for importing the clustering data.

## Results

**Figure 3.**
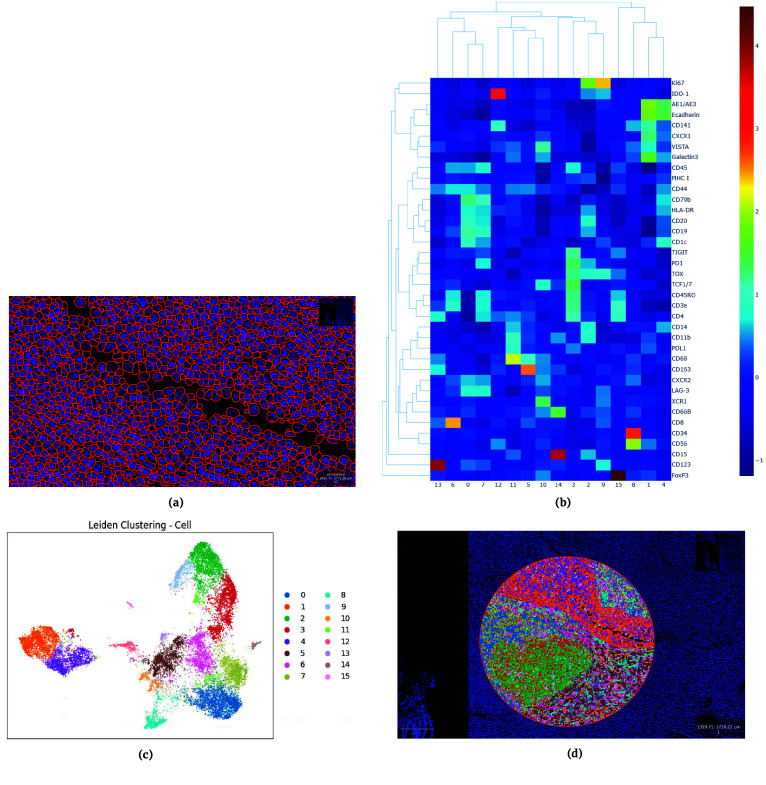
The analytic workflow was tested on tonsil tissue. (a) This is an image in QuPath illustrating the output from our containerized workflow on a tonsil region from a stained TMA image. Results of cell segmentation are shown as red cell outlines surrounding DAPI (blue) stained cell nuclei that define the cell data objects. (b) Results of Leiden unsupervised clustering of exported cell data from the segmented image as a heatmap. (c) Results of Leiden unsupervised clustering of exported cell data from the segmented image in reduced dimensions as a UMAP. (d) The results of importing clustering results back into QuPath and displaying it as a colormap over the original tonsil tissue image. Consistent color mappings allow comparison of cell type cluster within UMAP and spatial location of associated cells within the tissue image.

**Figure 4.**
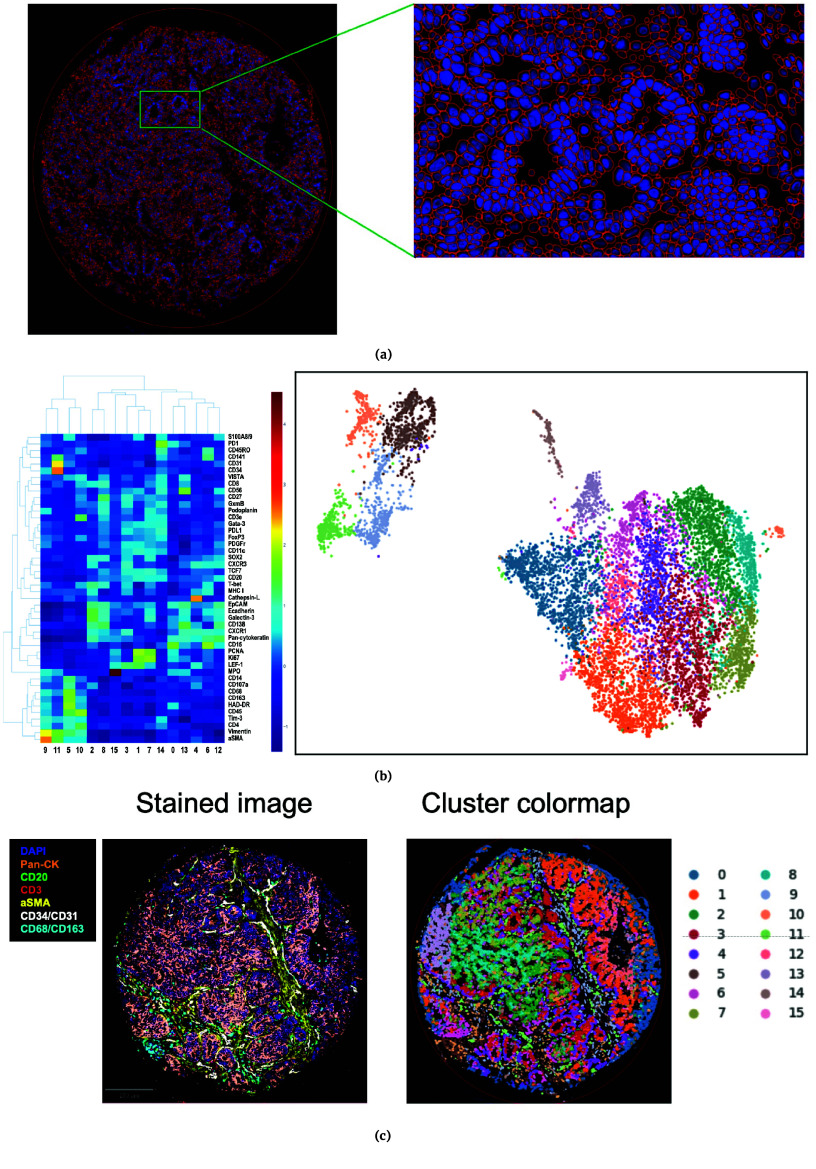
The analytic workflow was tested on human colorectal cancer tissue from a multi-cancer TMA. (a) This is an image in QuPath illustrating the output from our containerized workflow on a colorectal cancer region from a stained TMA image. Results of cell segmentation are shown as red cell outlines surrounding DAPI (blue) stained cell nuclei that define the cell data objects. (b) Results of Leiden unsupervised clustering of exported cell data from the segmented image as a heatmap (left) and as a UMAP (right). (c) The results of importing clustering results back into QuPath and displaying it as a colormap over the original colorectal cancer tissue image. Consistent color mappings allow comparison of cell type cluster within UMAP and spatial location of associated cells within the colorectal cancer tissue image.

This workflow was tested on tonsil (Figure 3) and human colorectal cancer (CRC) tissue (Figure 4) stained with PCF [11]. Our workflow yields cell segmentation consistent with visual inspection of segmentation results overlayed on stained images wherein each cell data units contained nucleus and cytoplasm for individual cells without significant over or under segmentation. Two pathologists reviewed the clusters in concert and annotated clusters of major cell types defined based on review of heat map relative marker expression and image overlays. To provide a preliminary quantitative assessment of segmentation quality, we manually reviewed StarDist segmentation results across three regions of interest in a tonsil TMA core (*n* = 285, 432, and 390 cells per region; 1,107 cells total). Incorrect calls, defined as non-cellular objects incorrectly segmented as cells or overlapping/oversegmented cell boundaries, totaled 56 across all regions, yielding an overall error rate of approximately 5.1%.

 In tonsil, cell types were found to appropriately co-express of markers for T cells (cluster 3,6,15), B cells (0,2,7), endothelial cells (cluster 8), macrophages (cluster 5,11), neutrophils (cluster 12), epithelial cells (1,4); (Figure 3b). For the CRC image, co-expression of markers defined T cells (cluster 10), macrophages (cluster 5), endothelial cells (cluster 11), stromal cells (cluster 9), as well as a large number of epithelial tumor clusters (0,2,4,6,8,12,13), showing the marked tumor heterogeneity in marker expression in this biopsy (Figure 4b). In UMAPs generated for both the tonsil and CRC tissues, we can see cell types grouped together is space that represent epithelial (and tumor) and stromal/immune compartments of the tissue (Figures 3c and 4b). Figure 3d shows example results of cluster colormap generation on regions of human tonsil through our containerized workflow showing the spatial location of cell types using the same color maps as employed in UMAPs for easy comparison. Figure 4c shows colormap generation of regions of the colorectal cancer image, with primary staining with select, cell type defining markers shown for comparison. Following cluster validation on the image, parameters could be interactively modified using the graphical user interface shown in Figure 2, and the analysis can be re-run, enabling reproducible and iterative analysis. Table 1 shows the antibody panel with target corresponding to the image from Figure 4.

**Table 1:** Antibody panel with target corresponding to the image in Figure 4.

Antibody	Target	Antibody	Target
aSMA	Smooth muscle/CAFs	CD31	Endothelial cells
CD123	pDCs	CD56	NK cells
CD14	Monocytes	CD66b	Neutrophils
CD163	M2 macrophages	CXCR1	Neutrophils
CD20	B cells	CXCR3	Th1/activated T cells
CD34	Endothelium/progenitors	Ecadherin	Epithelial cells
CD3e	T cells	Galectin-3	Macrophages/tumor
CD4	Helper T cells	GATA3	Th2/luminal cells
CD45	Leukocytes	GP100	Melanocytes/melanoma
CD45RO	Memory T cells	H2AX	DNA damage
CD68	Macrophages	HLA-DR	APCs
CD8	Cytotoxic T cells	IFN-g	Activated T/NK cells
EpCAM	Epithelial cells	Ki67	Proliferating cells
FoxP3	Tregs	LEF-1	T cells/Wnt-active
GzmB	Cytotoxic lymphocytes	MHC I	All nucleated cells
LAG-3	Exhausted T cells	MPO	Neutrophils
PanK	Epithelial cells	p16	Senescent/HPV+ cells
PCNA	Proliferating cells	PD-L1	Tumor/APCs
PD-1	Exhausted T cells	PDGFra	Fibroblasts
TIM-3	Exhausted T cells	Podoplanin	Lymphatic endothelium/CAFs
Cathepsin	Myeloid cells	S100A8/A9	Neutrophils/MDSCs
CD107a	Degranulating cells	SOX2	Stem-like/tumor cells
CD11b	Myeloid cells	T-Bet	Th1/cytotoxic cells
CD11c	Dendritic cells	TCF1/TCF7	Stem-like T cells
CD138	Plasma cells	TOX	Exhausted T cells
CD141	cDC1 dendritic cells	Vimentin	Mesenchymal cells
CD15	Neutrophils	VISTA	Myeloid/inhibitory
CD27	Memory lymphocytes		

We measured the execution time and memory requirements of processing human colorectal cancer (CRC) (shown in Figure 4). Our experiments were performed using GitHub Codespaces, with an instance with 4 cores, 16 GB of RAM and 32 GB of storage. The total execution time of processing a core is approximately 9 minutes.

## Conclusions

Image analysis workflows often consist of multiple steps, each of which runs in a different software tool that in turn require different dependencies and computing environments. This approach leads to major challenges in reproducibility, interoperability, and scalability, due to lack of systematic version control and local hardware. Moreover, most image analyses contain steps that are graphical and require interactive user input, features that are not supported by most workflow execution engines. We addressed these challenges by developing an open-source, containerized, graphical, and cloud-enabled spatial proteomics analysis workflow that easily integrates with other software through containerization.

**Figure 5.**
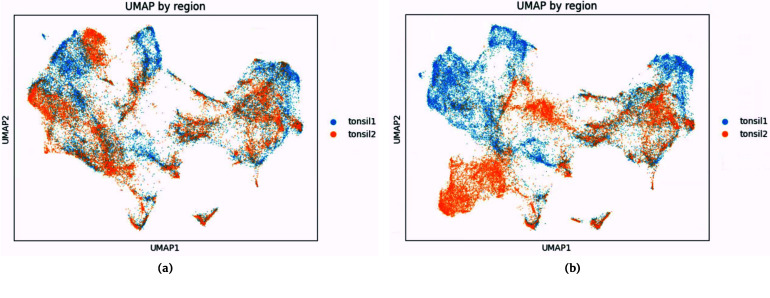
UMAP visualization of Leiden clustering results with and without the optional batch correction feature when two tonsil cores were selected in the test data uploaded to Zenodo. When multiple regions/images are selected, an additional UMAP colored by image sources or regions is generated as shown here. In this example, cells from the two tonsil cores appeared to partially overlap by visual inspection. (a) Leiden unsupervised clustering with batch correction. (b) Leiden unsupervised clustering without batch correction.

While our workflow was tested on tonsil and CRC tissues, this study is subject to limitations that represent opportunities for future work. First, a systematic quantitative assessment of StarDist against other segmentation methods using a fully annotated ground truth dataset was not performed. However, several studies in the literature, such as [12, 13] compared the performance of StarDist to other segmentation methods using annotated datasets. Second, we showed that the *optional* batch correction feature in our workflow can be turned on and off by showing results with and without batch correction (see Figure 5). There are many papers in the literature, such as [14, 15], that investigated the effectiveness and impact of sample size when batch correction was applied to harmonize image data across different sources that could give rise to variability. We acknowledge that when and how to apply batch correction to spatial proteomics data entails future work.

We integrated the QuPath image analysis tool as a containerized widget to automate our manual image analytical protocol consisting of cell segmentation, unsupervised clustering, and results visualization. Users can modify individual steps or widgets and corresponding input parameters using a drag-and-drop user interface. Additional modalities can easily be incorporated in this graphical workflow as additional widgets for specific types of spatial analysis desired, such as proximity values between individual cell types and cellular neighborhood analysis. Importantly, users can reproduce the work of others or incorporate additional analyses into their own workflows with ease. Finally, our workflows can be deployed locally, on internal computer servers or on the cloud such that data security and compliance can be directly managed by the user. This contrasts with commercial solutions in which sensitive patient data must be uploaded and analyzed on external proprietary server for which the user has limited control.

## Availability of source code and requirements

Lists the following:

- Project name: Graphical and containerized spatial proteomics workflow
- Project home page: https://github.com/BioDepot/SpatialProteomics
- Operating system(s): Ubuntu Linux, macOS (M-Series)
- Programming language: Python, Groovy, R
- Other requirements: Docker, Bwb
- License: MIT
- Available from bio.tools with ID biotools:spatial_proteomics and SciCrunch.org with RRID:SCR_027835
- Demonstration video: https://youtu.be/vHjdCAZQhfE
- A pre-configured cloud-based instance for testing is available at https://github.com/codespaces/new?hide_repo_select=true&ref=main&repo=979017184& skip_quickstart=true
- Available from WorkflowHub with DOI: https://doi.org/10.48546/WORKFLOWHUB.WORKFLOW.2015.4.

## Data Availability

The data set supporting the results of this article is available in the Zenodo repository with DOI: 10.5281/zenodo.15825670 [16]. This dataset contains 8 tissue cores including merkel cell carcinoma (MCC), breast carcinoma, squamous cell carcinoma (lung), hepatocellular carcinoma (HCC - liver), lung carcinoid, colorectal cancer (CRC), and 2 cores of tonsil.

## List of abbreviations

CODEX: co-detection by indexing; CRC: colorectal cancer CSV: comma-separated values; MFI: mean cell fluorescence pixel intensity; PCF: PhenoCycler-Fusion TMA: tissue microarray; UMAP: Uniform Manifold Approximation and Projections.
