# Supplementary material for: Graphical and interactive spatial proteomics image analysis workflow
Source: GigaByte. 2026 Jun 22;2026:gigabyte186. doi: 10.46471/gigabyte.186 (PMC13320228; doi:10.46471/gigabyte.186)
Supplement: Peer review history

# Reviewer report: Review MS


Reviewer name and names of any other individual's who aided in reviewerYanbo PanDo you understand and agree to our policy of having open and named reviews, and having your review included with the published manuscript. (If no, please inform the editor that you cannot review this manuscript.)YesIs the language of sufficient quality?YesPlease add additional comments on language quality to clarify if neededOverall clear and readable for a Technical ReleaseIs there a clear statement of need explaining what problems the software is designed to solve and who the target audience is? YesAdditional CommentsIs the source code available, and has an appropriate Open Source Initiative license <a href="https://opensource.org/licenses" target="_blank">(https://opensource.org/licenses)</a> been assigned to the code?YesAdditional CommentsAs Open Source Software are there guidelines on how to contribute, report issues or seek support on the code?YesAdditional CommentsIs the code executable?Unable to testAdditional CommentsThe manuscript provides a codespaces-based preconfigured instance and containerized workflow, which suggests executability; recommend adding a minimal “smoke test” section and expected outputs for verification.Is installation/deployment sufficiently outlined in the paper and documentation, and does it proceed as outlined?Unable to testAdditional CommentsA step-by-step “quickstart” in the manuscript (or clearly referenced in docs) would help users confirm deployment.Is the documentation provided clear and user friendly?YesAdditional CommentsIs there enough clear information in the documentation to install, run and test this tool, including information on where to seek help if required?YesAdditional CommentsIs there a clearly-stated list of dependencies, and is the core functionality of the software documented to a satisfactory level?YesAdditional CommentsHave any claims of performance been sufficiently tested and compared to other commonly-used packages? Not applicableAdditional CommentsThe manuscript shows qualitative outputs (segmentation, UMAP/heatmap, overlay) but lacks quantitative evaluation or benchmark vs existing workflows.Is test data available, either included with the submission or openly available via cited third party sources (e.g. accession numbers, data DOIs)?YesAdditional CommentsAre there (ideally real world) examples demonstrating use of the software? YesAdditional CommentsIs automated testing used or are there manual steps described so that the functionality of the software can be verified?Additional CommentsAny Additional Overall Comments to the AuthorThis Technical Release presents a useful, reproducible, and interactive spatial proteomics workflow integrating containerized QuPath with Scanpy-based Leiden clustering, ComBat batch correction, UMAP visualization, and re-import into QuPath for spatial validation. The engineering approach and availability of code/data/video/workflow records are strong. Major suggestions: (1) add minimal quantitative evidence for claims of accurate cell segmentation and biologically relevant clustering; (2) clarify when ComBat batch correction is appropriate and make it clearly optional, since region differences may reflect real biology; (3) improve reproducibility details by pinning key software/container versions and adding a simple smoke-test/expected-output verification section.RecommendationMinor Revisions


# Reviewer report: Review MS


Reviewer name and names of any other individual's who aided in reviewerRuijun TianDo you understand and agree to our policy of having open and named reviews, and having your review included with the published manuscript. (If no, please inform the editor that you cannot review this manuscript.)YesIs the language of sufficient quality?NoPlease add additional comments on language quality to clarify if neededIs there a clear statement of need explaining what problems the software is designed to solve and who the target audience is? NoAdditional CommentsIs the source code available, and has an appropriate Open Source Initiative license <a href="https://opensource.org/licenses" target="_blank">(https://opensource.org/licenses)</a> been assigned to the code?YesAdditional CommentsAs Open Source Software are there guidelines on how to contribute, report issues or seek support on the code?YesAdditional CommentsIs the code executable?YesAdditional CommentsIs installation/deployment sufficiently outlined in the paper and documentation, and does it proceed as outlined?Unable to testAdditional CommentsIs the documentation provided clear and user friendly?YesAdditional CommentsIs there enough clear information in the documentation to install, run and test this tool, including information on where to seek help if required?YesAdditional CommentsIs there a clearly-stated list of dependencies, and is the core functionality of the software documented to a satisfactory level?NoAdditional CommentsHave any claims of performance been sufficiently tested and compared to other commonly-used packages? Not applicableAdditional CommentsIs test data available, either included with the submission or openly available via cited third party sources (e.g. accession numbers, data DOIs)?YesAdditional CommentsAre there (ideally real world) examples demonstrating use of the software? YesAdditional CommentsIs automated testing used or are there manual steps described so that the functionality of the software can be verified?YesAdditional CommentsAny Additional Overall Comments to the AuthorThe manuscript presented an interactive and high-throughput automated spatial proteomics image analysis workflow that integrated two open-source tools (QuPath and CytoMAP) for cell segmentation, unsupervised clustering with batch correction, validation of clusters on the image, and cell type clustering results visualization, and all these analysis steps could be executed either end-to-end with a single click or run individually in an interactive manner. The authors employed a high-plex immunohistochemistry TMA containing diverse cancer and normal tissue types to demonstrate the broad applicability of this image analysis workflow. Overall, this work makes spatial proteomics image analysis more accessible and user‑friendly for the biomedical community. Given that this is submitted as a Technical Release to GigaByte, demonstrating computational robustness, performance metrics, and strict reproducibility is critical. Here are some comments to be considered. 1. Why were QuPath and CytoMAP chosen for integration? Were other open-source tools compared? Furthermore, to demonstrate the claim of being a "high-throughput" workflow, the manuscript completely lacks quantitative computational metrics and evaluation. Please report the total computational time (e.g., to process a standard image), memory/RAM requirements, and provide a brief benchmarking comparison against the existing pipelines mentioned in the introduction (e.g., Enable Medicine, SPEX, SPACEC). 2. Figure 3a only displayed DAPI staining results and the corresponding nuclear segmentation. It would be helpful to show the multiplex immunohistochemistry staining results. Additionally, the antibodies panel should be clearly described. 3. What do the labels 0-15 in Figure 3c represent? Which cell types correspond to each label? 4. The authors mentioned importing the clustering results back into QuPath for visualization. However, it remains unclear how Figure 3d demonstrates the consistency of the color masks based purely on visual inspection. Is there any quantitative metric available (e.g., spatial concordance, overlap score) to validate this consistency? 5. Although the authors employed a TMA containing multiple tumor types, representative multiplexed images and corresponding clustering results for each tissue type were not provided. Currently, Figure 3 only displays a single tonsil region. Including data for all mentioned cancer types is strictly necessary to validate the workflow's robustness and scalability across complex and diverse tissue architectures. 6. The authors should include relevant results from cell neighborhood clustering to further demonstrate the capability of their workflow, especially since "Cell neighborhood clustering" is explicitly listed as step 5 in the Figure 1 schematic. 7. The authors highlight the use of the ComBat algorithm to correct for region-specific batch effects. This is a critical feature; however, no data is presented to validate its efficacy. The authors must provide "Before Correction" and "After Correction" UMAP plots to demonstrate that the algorithm successfully eliminates technical batch effects while preserving true biological heterogeneity. Minor Concerns: 1. The abstract and Introduction suggest that CytoMAP is used for clustering analysis. However, the Implementation section states that clustering is actually performed using a Jupyter Notebook running the Leiden algorithm via the Python Scanpy package. Please clarify this significant discrepancy in the text and ensure the schematic workflow accurately reflects the true computational backend. 2. Figure 1 appears to be a low-quality screenshot, complete with visible spell-check indicators (typo markers) under some of the text. This is unsuitable for publication. Please replace this with a proper, high-resolution vector graphic or diagram. Importantly, the presentation of the manuscript need to be significantly improved.RecommendationMajor Revisions


# Reviewer report: Re-Review MS


Indicate in the comments box below whether you are happy with the changes made or if the manuscript is unacceptable.Comments on revised manuscriptThe authors have made a substantive and largely responsive revision. Most of the concerns raised in the previous round have been addressed in a reasonable and transparent manner. The revised manuscript now more honestly reflects what the workflow is: an end-to-end, containerized integration layer connecting QuPath, StarDist, and a Scanpy/Leiden notebook with a graphical widget interface, rather than a novel analytical method or a benchmarked high-throughput pipeline. This repositioning is appropriate and improves the scientific integrity of the paper. I recommend acceptance after minor revision. Several residual issues remain that should be addressed before publication. Residual Concerns (Minor) These do not block acceptance but should be addressed in the final version: 1, Quantitative segmentation validation is still absent. The authors replaced "accurate" with "consistent with visual inspection," which is honest, but the paper would benefit from even a small quantitative check — e.g., F1/IoU of StarDist segmentation against a manually annotated subset of cells in one ROI (n=200–500 cells would suffice). This is standard practice in the bioimage community and would strengthen credibility without major effort. If not added, the limitation should be explicitly acknowledged in the Discussion/Conclusions. 2, Batch-correction efficacy not demonstrated. Reviewer 2 specifically requested before/after UMAPs to validate ComBat. The authors' response — that no multi-region analysis is shown in the paper, therefore no batch correction was applied — sidesteps rather than addresses the question. Since ComBat is offered as a feature of the workflow, at least one supplementary figure showing its effect on a multi-region/multi-image case from the 8-core TMA they have now released would substantiate the feature. Without this, ComBat remains a documented option with no empirical demonstration of behavior within the manuscript itself. 3, "Pathologist review" is described qualitatively but without inter-rater detail. The text says "evaluated by 2 pathologists." It would strengthen the claim to state briefly whether the two pathologists reviewed independently or jointly, and whether there was disagreement on any cluster assignment. A single sentence suffices. 4, Figure 3b/4b heatmaps remain hard to read. Marker labels on the y-axis of the heatmaps are small and partially illegible at the rendered resolution. Given that the cluster annotations in the Results text (e.g., "cluster 3,6,15 = T cells") rely on these heatmaps for support, legibility is important. Consider increasing font size or providing a higher-resolution version. 5, Cell-neighborhood analysis removed from Figure 1. This is the correct fix for the discrepancy, but the Conclusions still mention "proximity values between individual cell types and cellular neighborhood analysis" as easily incorporable future modules. This is fine, but the authors should ensure no residual mention of neighborhood clustering exists elsewhere as a current feature. 6, Smoke test caveat. The note that "processing the downsampled image may not give the same outputs as processing the full-resolution original tissue image" is appropriate, but a smoke test whose output is not deterministically reproducible across users somewhat undermines its function as a verification mechanism. Consider providing a fixed expected-output hash or a deterministic seed for Leiden/UMAP so users can verify identical outputs, not just plausible ones.


# Editor report: Accept


Editor’s AssessmentThe manuscript is ready for formal acceptance.Editor’s AssessmentThe manuscript is ready for formal acceptance.
